# Profile of Enterobacteria Resistant to Beta-Lactams

**DOI:** 10.3390/antibiotics9070410

**Published:** 2020-07-15

**Authors:** Andressa Liberal Santos, Adailton Pereira dos Santos, Célia Regina Malveste Ito, Pedro Henrique Pereira de Queiroz, Juliana Afonso de Almeida, Marcos Antonio Batista de Carvalho Júnior, Camila Zanatta de Oliveira, Melissa Ameloti G. Avelino, Isabela Jubé Wastowski, Giselle Pinheiro Lima Aires Gomes, Adenícia Custódia Silva e Souza, Lara Stefânia Netto de Oliveira Leão Vasconcelos, Mônica de Oliveira Santos, Carla Afonso da Silva, Lilian Carla Carneiro

**Affiliations:** 1 Institute of Tropical Pathology and Public Health, Federal University of Goiás, 235 Street, Goiânia 74605-050, Brazil; andressa.liberal@gmail.com (A.L.S.); apsantos1906@yahoo.com.br (A.P.d.S.); crmalveste@hotmail.com (C.R.M.I.); pedrodequeiroz@gmail.com (P.H.P.d.Q.); juafonsoalmeida@gmail.com (J.A.d.A.); marcos260896@gmail.com (M.A.B.d.C.J.); larastefania@yahoo.com.br (L.S.N.d.O.L.V.); carlaafonsoufg@gmail.com (C.A.d.S.); 2 Agency of Agricultural Defense of Paraná—ADAPAR, Goioerê 80035-050, Brazil; cami.zanatta@hotmail.com; 3 Medicine College, Federal University of Goiás, 235 Street, Goiânia 74690-900, Brazil; melissa.avelino@uol.com.br (M.A.G.A.); mosbio21@gmail.com (M.d.O.S.); 4 Laranjeiras Unit, State University of Goiás, Goiânia 74705-010, Brazil; wastowski@gmail.com; 5 Department of Biology, Federal University of Tocantins, Square 109 North, NS15 Avenue, ALCNO-14-Plano Director North, Palmas 77001-090, Brazil; gipinheirolima@hotmail.com; 6 Department of Biology, Pontifical Catholic University of Goiás, 1st Avenue, Goiânia 74175-120, Brazil; lc-carneiro@hotmail.com

**Keywords:** antibiogram, antimicrobial resistance, β-lactams, enterobacterias, molecular diagnosis

## Abstract

A serious emerging problem worldwide is increased antimicrobial resistance. Acquisition of coding genes for evasion methods of antimicrobial drug mechanisms characterizes acquired resistance. This phenomenon has been observed in Enterobacteriaceae family. Treatment for bacterial infections is performed with antibiotics, of which the most used are beta-lactams. The aim of this study was to correlate antimicrobial resistance profiles in Enterobacteriaceae by phenotypic methods and molecular identification of 14 beta-lactamase coding genes. In this study, 70 exclusive isolates from Brazil were used, half of which were collected in veterinary clinics or hospitals Phenotypic methodologies were used and real-time PCR was the molecular methodology used, through the Sybr Green system. Regargding the results found in the tests it was observed that 74.28% were resistant to ampicillin, 62.85% were resistant to amoxicillin associated with clavalunate. The mechanism of resistance that presented the highest expression was ESBL (17.14%). The genes studied that were detected in a greater number of species were *bla*GIM and *bla*SIM (66.66% of the samples) and the one that was amplified in a smaller number of samples was *bla*VIM (16.66%). Therefore, high and worrying levels of antimicrobial resistance have been found in enterobacteria, and a way to minimize the accelerated emergence of their resistance includes developing or improving techniques that generate diagnoses with high efficiency and speed.

## 1. Introduction

Regarding taxonomy, in relation to taxonomy, the Enterobacteriacea family has 53 genera of which more than 170 species have already been named. Among these, 26 bacterial genera have already been associated with bacterial infections in humans. Members of this family are Gram-negative, facultative anaerobic rods and most species are able to grow at 37 °C, although some grow more properly at 25 to 30 °C [1]. 

These microorganisms are widely distributed in nature and are found in soil, water, vegetables, in humans and vertebrates gastrointestinal tract [2]. Enterobacteriaceae represent the main group of bacteria isolated in clinical samples and are associated with a wide variety of community and hospital infections [3]. Gram-negative bacteria, specifically Enterobacteriaceae, are common causes of both community-acquired and hospital-acquired infections, including urinary tract, bloodstream, and lower respiratory tract infections [4].

Resistance among clinically important organisms to antimicrobial agents is severely threatening the repertoire of treatment options for common infections. The challenge is intensified by the fact that several of these organisms are resistant to multiple antimicrobials [5]. Infections caused by Gram-negative bacteria resistant to multiple drugs are a serious public health problem due to the scarcity of treatment options for these infections [6]. 

Currently, antimicrobial resistance is one of the most important factors that threaten public health [7]. Transmission between species of resistant bacteria or genetic elements of resistance from animals or the environment to humans has been reported [8,9]. Monitoring hospital environments and those related to animal husbandry and treatment has permanently entered the timeline of the most important studies and annual reports that assess the scope and level of this phenomenon [10].

Antibiotics play a key role in the success of some medical practices. Unfortunately, they tend to lose their efficacy over time due to the emergence and spread of resistance among bacterial pathogens [11]. According to Magiorakos [12], bacteria can be multi drug-resistant (MDR). MDR was classified as having acquired non-susceptibility to at least one agent in three or more antimicrobial categories.

Drug resistance genes can be spread from one bacterium to another through various mechanisms such as plasmids, bacteriophages, naked DNA or transposons. Some transposons contain integrons—more complex transposons that contain a site for integrating different antibiotic resistance genes and other gene cassettes in tandem for expression from a single promoter [13]. Bacterial conjugation is the most sophisticated form of horizontal gene transfer (HGT) in bacteria and provides a platform for the spread and persistence of antibiotic resistance and virulence genes [14]. 

Beta-lactams are preferred because of their clinical efficacy and safety by virtue of their highly selective toxicity [15]. Resistance to beta-lactams in Enterobacteriaceae and other Gram-negative organisms is primarily mediated by beta-lactamases [16]. Beta-lactamases are enzymes that catalyze the beta-lactam hydrolysis ring leading to antimicrobial inactivation and preventing it from being active against the enzymes responsible for bacterial cell wall synthesis [17].

Antimicrobial susceptibility testing methods are divided into types based on the principle applied in each system [18]. The antibiogram provides qualitative results by categorizing bacteria as susceptible, intermediate susceptibility or resistant. Therefore, it is a tool based on the resistance phenotype of the tested microbial strain. However, inhibition of bacterial growth does not mean bacterial killing, the phenotypic method fails to distinguish between bactericidal and/or bacteriostatic effects [19].

Molecular diagnosis is another method of identifying bacterial resistance that can be applied. The molecular technique performed through nucleic acids, while requiring advancements, may allow the patients to obtain a fast examination result, within a four-hour period; thus, initiating the most appropriate antibiotic therapy. This can improve treatment outcomes for the patient and reduce empirical antimicrobial prescriptions, decreasing the duration and cost of antimicrobial treatment. Thus, technologies with the diagnosis of nucleic acids have the potential to reduce the selection of new resistances as well as to reduce the potential of existing resistances [20].

The objectives of this study are to correlate the resistance profiles of Enterobacteria using phenotypic and genotypic methodologies. The genes encoding resistance to beta-lactams are: *bla*SPM, *bla*SIM, *bla*VIM, *bla*KPC, *bla*SHV, *bla*CTX-M, *bla*GIM, *bla*OXA, *bla*IMP, *bla*NDM, *bla*SME, *bla*DHA, *bla*CMY and *bla*TEM. This study is justified because it is assumed that molecular methods improve accuracy and efficiency compared to the classical phenotyping method. In addition, it can be released in a short time; helping to improve the effectiveness of antibiotic therapy.

## 2. Results

The enterobacteria from this study were isolated from samples collected in four different types of origin. Among the total bacterial isolates, 40% are from the human clinic, 20% from an animal clinic, 10% from a human hospital environment and 30% from the veterinary hospital environment.

This antimicrobial resistance study in enterobacteria characterized a phenotypic profile of resistance to beta-lactam antibiotics, in which among the 70 bacterial samples studied, 52 (74.28%) were resistant to ampicillin, 44 (62.85%) were resistant to amoxicillin associated with the beta-lactamase inhibitor clavalunate, 38 (54.28%) were resistant to cefazolin, and 6 (8.57%) were resistant to cefuroxime. Table 1 shows the percentage of antimicrobial resistance by sample source of Enterobacteriaceae.

Phenotypically, using the antibiogram method, *Enterobacter aerogenes*, *Enterobacter agglomerans* and *Cedecea neteri* species stand out, which have the highest resistance rate, being resistant to 10 of the 11 tested antibiotics (90.9%). 

**Figure 1:**
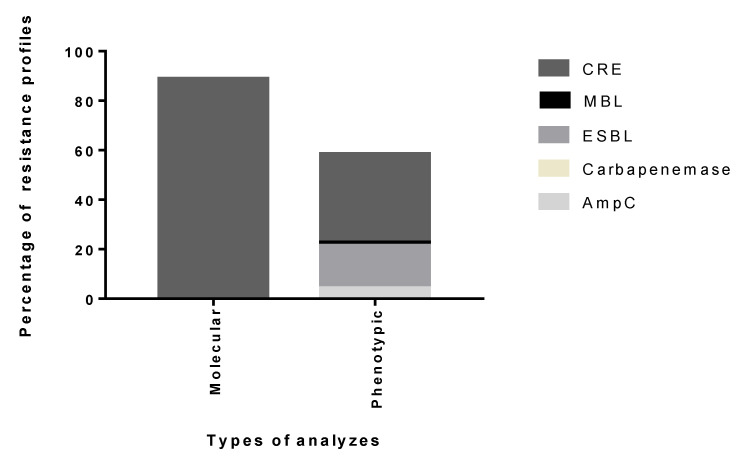
Percentage of profiles of resistance in Enterobacteriaceae.

The sensible and resistance profiles, found phenotypically in this study, determined that among the enterobacteria studied here there was a predominance of 2.8% sensible and 97.2% resistance profiles. Among the resistance profiles, potential MDR profiles were also researched and ESBL, AmpC, MBL, Carbapenemases and CRE were quantified. These data can be observed in Figure 1. 

Phenotypic analyzes revealed that 28.5% of the total bacteria studied are MDR. Still on the phenotypic profiles, the penicillin group was the antibiotic for which there was the highest resistance rate. There was resistance in at least one of the studied penicillins, in approximately 85.71% of the bacteria. For cephalosporins, there was resistance to at least one of those tested in 77.14% of bacteria, a relatively high number of which shows that these drugs, from the first to the fourth generation, are also losing their effect on enterobacteria. 

In relation to β-lactams used as drugs of last resource–carbapenems–in this study, there was phenotypic resistance to imipenem in 35.71% of the isolated bacteria determining the CRE profile. The antibiotic that presented the lowest percentage of resistance was the monobactam aztreonam with a resistance rate of 34.28%, which corresponds to a rate close to that of carbapenems, showing that these antibiotics were the most effective against most bacterial samples studied.

The species *E. agglomerans* showed phenotypic resistance data with profiles sensitive, MDR and CRE. This shows that within the same bacterial species the resistance possibilities are very variable. The species *Yersinia ruckeri*, was the one that presented more number of resistance mechanisms (ESBL, MBL and AmpC). The species that did not present any of the mechanisms were: *Escherichia blattae*, *Hafnia alvei*, *Raoultella terrigena* and *Citrobacter freundii*. 

The percentages of amplification of the beta-lactamase genes, through the qPCR method, found among Enterobacteriaceae were: 66.66% for the *bla*GIM and *bla*SIM genes, 61.11% for the *bla*DHA and *bla*TEM genes, 55.55% for the *bla*CMY, *bla*CTX-M, *bla*NDM, *bla*OXA genes, 50% for the *bla*IMP gene, 44.44% for the *bla*SHV and *bla*SPM genes, 38.88% for the *bla*KPC gene, 33.33% for the *bla*SME gene and 16.66% for the *bla*VIM gene. 

The fenotypic profile of resistance to beta-lactam antimicrobials was determined in 19 bacterial species among enterobactérias: regarding all the phenotypic resistances found, the species *E. aerogenes*, *E. agglomerans*, *C. freundii* and *C. neteri*, stood out showing the highest resistance rate (90.9%). For methodological reasons the resistance mechanisms ESBL, AmpC, MBL and Carbapenemases have not been studied molecularly, only CRE. The molecular profile of resistance to beta-lactam antimicrobials was determined in 18 bacterial species among enterobacteria: 94.44% showing resistance for aztreonam, ceftazidima, cefoxitin and piperacillin associated with beta-lactamase inhibitor tazobactam. Phenotypic and molecular data are compared in Table 2.

By analyzing the amplification rate of the genes that confer beta-lactam resistance and making an association of the same with the literature review carried out in this study, it was observed that the species that showed potential resistance to a greater number of antibiotics were: E *aerogenes*, *R. terrigena*, *Moganella morganii*, *Edwardsiella ictaluri*, *C. neteri*, *Salmonella paratyphi* and *Y. ruckeri*., exhibiting resistance potential for all antibiotics (100%) tested. *C. freundii*, *Klebsiella* spp., *E. coli*, *E blattae*, *Providencia rustigiani and Erwinia persicina* were studied, showing potential resistance in 10 among 11 antibiotics (90.9%) tested.

The resistance information obtained in this study shows that 100% of the analyzed species present a high potential for resistance to several beta-lactams. Among the potential profiles suggested by the qPCR analyzes, the MDR and CRE data were, respectively, 100% and 88.88% among the studied species. These data can be observed in Figure 1. 

The Pearson coefficient was calculated to linearly correlate two variables. The Pearson correlation coefficient varies between −1 and 1. The signal indicates the direction of correlation (negative or positive) while the value indicates the magnitude. The closer to 1 the stronger the level of linear association between variables 3. 

In this study, the detection rate of antimicrobial resistance by molecular methodology was generally higher than the detection rate by phenotypic methodology. This study showed that the presence of the resistance gene in the bacterial genome does not necessarily imply its expression, therefore it is necessary to develop the phenotypic methodology.

An experiment was carried out to verify the plasmid profile and from plasmid DNA digested were no identified sites to *Eco*R I and *Hind* III restriction enzymes. After these results, the authors decided that the best experiment to observe the restriction plasmid profile must be the sequencing experiments that will be done in another study.

## 3. Discussion

The phenotypical results of the present study are in agreement with a retrospective study that was carried out in a laboratory of clinical analyzes of Goiânia, Goiás, which evaluated the prevalence and antimicrobial susceptibility profile of the isolated microorganisms from 432 samples, in which the species *E. aerogenes*, *E. agglomerans* and *C. neteri* are related among those that present resistance to multiple drugs [21]. 

Except for *C. neteri*, which is a strain of animal origin, these bacterial species are in accordance with epidemiological data indicated by ANVISA [22], as they are among the species of enterobacteria most prevalent in primary bloodstream infections associated with the use of catheters in hospitalized patients in adult, pediatric and neonatal ICUs in Brazil.

Regarding multidrug resistance (MDR) and carbapenem-resistant Enterobacteriaceae (CRE) profiles have recently been updated by the Center for Disease and Control and European Center for Diseases Control and Prevention, promoted aiming at the international standardization of these terminologies, as published by Magiorakus et al, [12], in which, MDR was defined as the resistance to at least one agent in three or more categories of antimicrobials. 

According to the authors, Enterobacteria resistant to carbapenems infections are associated with high mortality rates (up to 70%), making them particularly challenging from a clinical standpoint [11].

Logan and Weinsteins [21] showed a global distribution of carbapenemase degenerations in Enterobacteriaceae. Carbapenem-resistant enterobacteria have emerged as a major cause of nosocomial infections worldwide and are characterized by rapid and progressive dissemination [23].

In a study of CRE isolated from patients who received medical care at Stanford Health Care and Lucille Packard Children’s Health, California, USA, between January 2013 and December 2016, carbapenem minimum inhibitory concentration (MICs) for the CRE card ranged from ≤1 to 265 >8 μg/mL for imipenem, which also demonstrated higher resistance to this antimicrobial [24]. 

A study carried out in northeastern Brazil showed that in 672 positive urocultures for urinary tract infection, the etiological agent belonged to the Enterobacteriaceae family in 86.9%, and among them 29 (4.8%) were ESBL [25].

A literature review was carried out to determine the potential of resistance by molecular methodology found in this study, which has genes described in the literature that encode the β-lactamase enzyme. The result of the literary survey is shown in Table 3 [3,6,16,26,27,28,29,30,31,32,33,34,35,36,37,38,39,40,41,42,43,44,45,46,47,48,49,50,51,52,53,54,55,56,57,58,59,60,61,62,63,64,65,66,67,68,69,70,71,72,73,74,75,76,77,78,79,80,81,82,83,84,85,86,87,88,89,90,91,92,93,94,95,96,97,98,99,100,101,102,103,104,105,106,107,108,109,110,111,112,113,114,115,116,117].

The data from the bibliographic review carried out here show that the beta-lactamases most frequently correlated with beta-lactams are *bla*OXA, *bla*TEM, *bla*KPC, *bla*CTX-M and *bla*SHV. However, among the total genes found in this study, the ones with the highest percentage were *bla*GIM and *bla*SIM, both with 66.66%. In the literature review, the *bla*GIM gene was correlated only with beta-lactam imipenem, while *bla*SIM was correlated with ampicillin, aztreonam, ceftazidime, cefepime, imipenem and piperacillin + tazobactam. Resistance to beta-lactam imipenem gives the bacteria the CRE profile, and although phenotypic analyzes show a low detection rate of imipenem (35.71%), the molecular detection rate of imipenem was the second-highest detection rate with a value of 88.88%.

In a study carried out with clinical isolates of carbapenem-resistant Enterobacteriaceae collected at the University Hospital of Santa Maria, Rio Grande do Sul, Brazil, the *bla*KPC, *bla*OXA-48, *bla*NDM, *bla*SPM, *bla*IMP, *bla*VIM and *bla*GIM genes were investigated by PCR and multiplex PCR. About the number of studied microorganisms, the genotypic tests evidenced that *bla*KPC was the most prevalent gene, in 31% (*n* = 10) of the samples, followed by *bla*IMP, in 12.5% (*n* = 4) [118].

In a study conducted in eight hospitals in Paris surroundings, France, twelve isolates were collected in twelve patients, 11 *Klebsilla pneumoamiae* and 1 *Klebsilla oxytoca*. All isolates showed *bla*DHA gene and (4/12) 33.33% *bla*TEM gene [119].

In another study, 88 phenotypically ESBLs positive isolates samples collected from hospitals located in Mizoram, India, enterobacteria such as *E. coli*, *K. pneumoniae* and *Salmonella* spp. were isolated. All the isolates were tested for the presence of *bla*CTX-M-1 and/or *bla*SHV genes by PCR assay. A total of 54 (13.04%) isolates carried at least one ESBLs genes tested under this study, of which 41 (9.90%) *E. coli*, 11 (2.66%) *K. pneumoniae* and 2 (0.48%) *Salmonella* were found to be positive for *bla*CTX-M-1/*bla*SHV gene. A total of 4 (10.14%) and 9 (2.17%) isolates were positive for *bla*CTX-M-1 and *bla*SHV genes, respectively, whereas, 3 (0.72%) *K. pneumoniae* isolates were positive for both the genes. On the other hand, only 2 (0.48%) *Salmonella* isolates for *bla*CTX-M-1 gene [120].

In our study, both rates are lower than those found in the phenotypic profile, however, it should be considered that the molecular analysis was performed in only 18 representatives of the studied species, thus presenting a smaller sample than the phenotypic tests. This fact explains why the data from the molecular analyzes are denominated only as potential and also, in addition, the presence of genes in the genome does not necessarily imply phenotypic expression of them [121].

Even molecular analyzes genes are not expressed as host carriers and the only fact of being present in circulating strains is already a high risk, since the onset and spread of the microorganism with drug resistance shows the problem of the interaction of several factors such as an exchange of genetic information between microorganisms, through the transfer of genes to new hosts [32]. 

The presence of more resistance profiles in molecular analyzes than in phenotypic analyzes testify the greater sensitivity of the molecular methodology. qPCR provides a high advantage of fast transferring detection rate and quantification of target DNA sequences in different matrices. The low amplification time is facilitated by the simultaneous amplification and visualization of the new amplicons formed. However, the mere presence of genes responsible for components of antimicrobial resistance or toxin production does not automatically signify their expression or production [34]. Thus, although molecular techniques are very useful, particularly for rapid results, they should be confirmed with standard phenotypic sensitivity tests [122].

The statistical method was performed [123], and according to the low linear correlation found in this study (r^2^ = 0.0015 or r = 0.038), it should be known as a comparative analysis of the efficiency of the two methodologies for the detection of antimicrobial resistance. The molecular methodology, PCR, is appreciated due to its high capacity of sensitivity and specificity [124]. The low linear correlation found with the Pearson coefficient in this study evidences limitations of the phenotypic methodology and shows greater sensitivity of the molecular methodology for the detection of antimicrobial resistance.

However, it should be bear in mind the conditions offered in the growth medium diverge from the actual conditions of a host organism. Since the growth medium is a favorable environment for bacterial growth, it offers optimal conditions for bacterial metabolism, a fact that does not occur in the host organism. This variation of conditions may be determinant for gene regulation, generally leading to the expression of a greater number of genes in the environment of metabolic stress or gene suppression in an environment with favorable growth conditions. This explains why the molecular data found here is compatible with epidemiological data [125].

## 4. Methods

A total of 70 bacterial samples of Enterobacteriaceae were stored in a bio-repository at the Laboratory. Among the analyzed bacteria are the species: *Klebsiella pneumoniae, Proteus mirabilis*, *Citrobacter freundii*, *Morganella morgani*, *Providencia* spp., *Enterobacter aerogenes*, *Enterobacter agglomerans*, *Raoultella terrigenes*, *Escherichia coli*, *Escherichia blatteae*, *Edwarsiella ictaluri*, *Cedecea neteri*, *Erwinia persicina*, *Providencia rustigiani*, *Salmonella paratyphia*, *Salmonella typhi*, *Yersinia ruckeri*, *Serratia marcecens* and *Hafnia alvei* (Table 4). Bacteria came from mucosa of human tonsils (five samples), human corneas (twenty-three samples), animal bladder (four samples), animal uterus (ten samples), Veterinary Hospital Environment (twenty-one samples) as well as respiratory equipment from a hospital service, Manual Resuscitators-MRI (seven samples). The isolates of human tonsils were from Hospital of the clinics of the Federal University of Goiás, Brazil; human corneas from the Service of Verification of Deaths (SVO) of Goiânia, Goiás, Brazil; the animal bladder and uterus samples were obtained from a female dog hospitalized in a veterinary hospital of Goiânia, Goiás, Brazil; veterinary hospital environment samples came from the Dog Center clinic in Goiânia, Goiás, Brazil and manual resuscitators from an Intermediate Care Unit (ICU) of a public hospital in the state of Tocantins, Brazil. 

After being stored as a biorepository, these enterobacteria were randomly used in this study to compare the resistance profile presented by both phenotypic and genotypic methodology.

The antibiogram and sensitivity of the Gram-negative bacilli samples to the various antimicrobials were performed according to agar-diffusion methodology (Kirby-Bauer), according to the bacterial genus were used the antimicrobials ampicillin 30 μg, amoxiline-clavulanate 20/10 μg, aztreonam 30 μg, cefazolin 30 μg, cefepime 30 μg, cefoxitin 30 μg, cefuroxime 30 μg, ceftazidime 30 μg; ceftriaxone 30, imipen 10 μg and piperacillin-tazobactam 100/10 μg. The quality control procedure was followed, strains *E. coli* ATCC^®^ 35,218 were used for combinations of β-lactam inhibitors/β-lactamases [126]. 

For the phenotypic detection of extended spectrum beta-lactamases (ESBL) production, from enterobacteria of this study, the statistic method was performed [126] and according to the results low linear correlation was found (r2 = 0.0015 or r = 0.038), and it is important to recognize that the data presented good efficiency in both methodologies for the detection of antimicrobial resistance. 

For the AmpC-type beta-lactamase phenotypic detection, the induction test was performed using antimicrobial susceptibility testing, performed by the disk diffusion assay (Kirby–Bauer technique) according to the 2015 European Committee on Antimicrobial Susceptibility Testing (EUCAST) recommendations [127].

Imipenem and meropenem discs were used for the carbapenemases phenotypic investigation and interpretation of the sensitivity following the criteria established by CLSI [128]. At least one bacterium that showed resistance of the carbapenems was submitted to the MBL screening test, using the enzyme blockade method and following the recommendations of ANVISA [22]. The test used imipenem (10 μg) and meropenem (10 μg) disc, positioned parallel to two other imipenem and meropenem discs added with 10 μL of EDTA. 

For enterobacteria, in addition to the EDTA test, the modified Hodge test (MHT) was also performed. MHT consists of the inoculation of *E. coli* ATCC 25922^®^ on the entire surface of a Müller-Hintos agar plate. A meropenem disk was placed in the center of the plate and around this disk streaks were made with the suspected samples, as recommended by CLSI [128].

For each bacterium, plasmid extraction was done according to the FLEXIPREP extraction kit manual from Pharmacia^®^, according to the manufacturer’s instructions. For the qPCR assays, specific primers were designed based on the sequences deposited in GenBank (Table 5).

Reactions were prepared using the Sybr Green (Sybr Green qPCR master mix LOW ROX-100 reactions × 25 uL) real-time PCR kit, following the methodology suggested by the manufacturer. For the positive and endogenous control of the reaction the primers were used to amplify the 16S RNA, for the negative control, water was added in place of the DNA. Fisher’s test was used to compare the techniques considering isolated samples.

Purified plasmid DNA preparations were digested with restriction enzymes for identification and characterization of the genes of that study according to the preparation: in microcentrifuge tubes were added: 2 μL of 10× Buffer (Ludwigbiotec), (Buffer EcoR I for enzyme *Eco*R I and Buffer V2 for *Hind* III); 1 μL of *Eco*R I or *Hind* III enzyme (10 UI/μL) (Ludwigbiotec), 15 μL H2O; 2 μL template DNA (~300 ng/μL). The tubes were placed in thermo-blocks at 37 °C overnight and were then incubated at −20 °C for 15 minutes. From these preparations agarose gel electrophoresis was performed, as controls were used the preparation without the enzyme and a non-incubated preparation.

## 5. Conclusions

This study demonstrated high resistance levels of enterobacteria to various antimicrobials, both in humans and animals. The present antimicrobial resistance study characterized phenotypic and molecular profiles of resistance to beta-lactam antibiotics in enterobacteria. The phenotypic profile was demonstrated by the Antimicrobial Sensitivity Test, performed by plate-diffusion (antibiogram), while the molecular profile was demonstrated from the Molecular Resistance Potential analyzes, which associates data from the literature review to the amplification by quantitative PCR. MDR and CRE profiles were found. In this characterization, the detection rate by molecular methodology was higher, demonstrating the greater sensitivity of this technique.

According to the results obtained here, it can be determined that, given the need for faster diagnosis in emergencies or not, the molecular method, because its more sensitive, faster and less laborious process, can be considered superior to the phenotypic method in which has some limitations such as dependence on specific conditions of reproduction for the ideal growth of bacteria, detection of only cultivable organisms, previous preparation of the material, greater manipulation and risk of contamination, and longer time for the final diagnosis.

## Figures and Tables

**Table 1:** Percentage of antimicrobial resistance by sample source (%).

Antibiotics	Percentage of Antimicrobial Resistance (%)					
	Manual Resuscitators	Human Cornea	Human Tonsilas	Veterinary Hospital	Animal Bladder	Animal Uterus
Ampicillin	42.85	65.21	0.2	90.47	100	100
Aztreonam	0	26.08	0	85.71	0	0
Amoxicillin-clavalunate	0	39.13	0	90.47	100	100
Ceftazidime	100	30.43	0	85.71	100	0
Cefoxitin	42.85	39.13	0.2	76.19	0	0
Cefazolin	0	52.17	100	80.95	100	0
Cefepime	100	30.43	0	95.23	0	0
Ceftriaxone	0	26.08	0	0	0	0
Cefuroxime	100	26.08	0	76.19	0	0
Imipenem	100	30.43	0.2	28.57	100	0
Piperacillin-tazobactam	100	17.39	0	67.66	100	0

**Table 2:** Rates of detection of phenotypic and molecular antimicrobial resistance.

Antimicrobials	Molecular Detection Rate (%)	Phenotypic Detection Rate (%)	Descriptive Statistics		
			Standard Deviation	Default Error	Variance
Ampicillin	83.33	74.28	6.39931637	4.525	40.95125
Aztreonam	94.44	34.28	42.53954396	30.08	1809.6128
Amoxicilina + Clavalunate	88.88	62.85	18.40599	13.015	338.7805
Ceftazidime	94.44	51.42	30.41973	21.51	925.3602
Cefoxitine	94.44	41.42	37.4908	26.51	1405.56
Cefazoline	38.88	54.28	10.88944	7.7	118.58
Cefepime	88.88	44.28	31.53696	22.3	994.58
Ceftriaxone	88.88	41.42	33.55929	23.73	1126.226
Cefuroxime	72.22	8.57	45.00735	31.825	2025.661
Imipenem	88.88	35.71	37.59687	26.585	1413.524
Piperacillin + Tazobactam	94.44	41.42	37.4908	26.51	1405.56

**Table 3:** Bibliographical survey concerning the phenotypic resistance of beta-lactamases against the corresponding resistance genes. subtitle: the (+) sign indicates a correlation in the literature of the corresponding beta-lactamase coding gene of the column, with the corresponding antibiotic in the horizontal line. while the (-) sign indicates an absence of correlation in the gene and antibiotic literature.

Antibiotics	*bla*_OXA_	*bla*_IMP_	*bla*_NDM_	*bla*_SME_	*bla*_DHA_	*bla*_CMY_	*bla*_TEM_	*bla*_KPC_	*bla*_SPM_	*bla_CTX-M_*	*bla*_VIM_	*bla*_SIM_	*bla*_GIM_	*bla*_SHV_
Ampicillin	+	-	-	+	-	+	+	+	+	+	-	+	-	+
Aztreonam	+	+	+	-	+	+	+	+	+	+	+	+	-	+
Amoxicillin + Clavanulate	+	+	+	-	-	+	+	+	-	+	+	-	-	+
Ceftazidime	+	+	+	-	+	+	+	+	+	+	+	+	-	+
Cefoxitin	+	-	+	-	+	+	+	+	+	+	-	-	-	+
Cefazolin	+	+	+	-	+	+	+	+	-	+	-	-	-	+
Cefepime	+	+	+	-	+	+	+	+	+	+	+	+	-	+
Ceftriaxone	+	+	+	-	+	+	+	+	-	+	-	-	-	+
Cefuroxime	+	-	+	-	-	-	+	+	+	+	+	-	-	+
Imipenem	+	+	+	+	+	+	+	+	+	+	+	+	+	+
Piperacillin + Tazobactam	+	+	+	-	-	+	+	+	+	+	+	+	-	+

**Table 4:** Quantification of studied bacterial genera.

Bacterial Genus	Number of Samples
*Cedecea neteri*	2
*Citrobacter freundii*	3
*Edwardsiella ictaluri*	1
*Enterobacter aerogenes*	12
*Enterobacter agglomerans*	6
*Erwinia persicina*	1
*Escherichia blattae*	1
*Escherichia coli*	12
*Hafnia alvei*	3
*Klebsiella* spp.	7
*Morganella morganii*	2
*Proteus mirabilis*	4
*Providencia rustigiani*	1
*Providencia* spp.	1
*Raoultella terrigena*	1
*Salmonella paratyphia*	1
*Salmonella* spp.	2
*Salmonella typhi*	2
*Serratia marcecens*	6
*Yersinia ruckeri*	1
*Yersinia* spp.	1

**Table 5:** Oligonucleotides used for amplification of the β-lactam resistance genes of this study.

Genes	Gene Sequence from 5′ to 3′	Temperature of Ringing	Quantity of Bases	Access at the GenBank	Amplified Fragment Size
*bla*OXA	Sense: GGCAGCGGGTTCCCTTGTC	49.7	19	FN396876.1	171pb
	Reverso: CGATAATGGGCTGCAGCGG	49.7	19		
*bla*IMP	Sense: CCAGCGTACGGCCCACAGA	49.6	19	NG035455.1	138pb
	Reverso: GGTGATGGCTGTTGCGGCA	50.3	19		
*bla*NDM	Sense: CGGCCGCGTGCTGGTG	49.8	16	JN711113.1	182pb
	Reverso: GGCATAAGTCGCAATCCCCG	50.2	20		
*bla*SME	Sense: GGCGGCTGCTGTTTTAGAGAGG	50.9	25	KJ188748.1	184pb
	Reverso: TGCAGCAGAAGCCATATCACCTAAT	50.3	22		
*bla*DHA	Sense: GCGGGCGAATTGCTGCAT	49.8	18	NG041043.1	183pb
	Reverso: TGGGTGCCGGGGTAGCG	50.1	17		
*bla*CMY	Sense: GGATTAGGCTGGGAGATGCTGAA	50.1	23	NG041279.1	158pb
	Reverso: CCAGTGGAGCCCGTTTTATGC	49.6	21		
*bla*TEM	Sense: TCCGTGTCGCCCTTATTCCC	49.6	20	KJ923009	165pb
	Reverso: CCTTGAGAGTTTTCGCCCCG	49.6	20		
*bla*SHV	Sense: GGCAGCGGGTTCCCTTGTC	49.7	19	FN396876.1	171pb
	Reverso: CGATAATGGGCTGCAGCGG	49.7	19		
*bla*VIM	Sense: GTTATGCCGCACCCACCCC	50.3	19	NG036099.1	194 pb
	Reverso: ACCAAACACCATCGGCAATCTG	49.7	22		
*bla*SPM	Sense: CGAAAATGCTTGATGGGACCG	50.3	21	DQ145284.1	147pb
	Reverso: CACCCGTGCCGTCCAAATG	49.7	19		
*bla*CTX	Sense: CTGAGCTTAGCGCGGCCG	50.1	18	FJ815279.1	189pb
	Reverso: AATGGCGGTGTTTAACGTCGG	50.0	21		
*bla*GIM	Sense: CGGTGGTAACGGCGCAGTG	50.2	19	JX566711.1	149pb
	Reverso: TGCCCTGCTGCGTAACATCG	50.2	20		
*bla*KPC	Sense: GGCGGCTCCATCGGTGTG	49.5	18	AF297554.1	155pb
	Reverso: GTGTCCAGCAAGCCGGCCT	50.4	19		
*bla*SIM	Sense: GCACCACCGGCAAGCGC	50.8	17	EF125010.1	156pb
	Reverso: TGTCCTGGCTGGCGAACGA	50.0	19		
